# Bonding With Bot: User Feedback on a Chatbot for Social Isolation

**DOI:** 10.3389/fdgth.2021.735053

**Published:** 2021-10-06

**Authors:** Gilly Dosovitsky, Eduardo L. Bunge

**Affiliations:** Psychology Department, Palo Alto University, Palo Alto, CA, United States

**Keywords:** chatbot, mental health, social isolation, user feedback, thematic analysis, qualitative – quantitative analysis

## Abstract

Social isolation has affected people globally during the COVID-19 pandemic and had a major impact on older adult's well-being. Chatbot interventions may be a way to provide support to address loneliness and social isolation in older adults. The aims of the current study were to (1) understand the distribution of a chatbot's net promoter scores, (2) conduct a thematic analysis on qualitative elaborations to the net promoter scores, (3) understand the distribution of net promoter scores per theme, and (4) conduct a single word analysis to understand the frequency of words present in the qualitative feedback. A total of 7,099 adults and older adults consented to participate in a chatbot intervention on reducing social isolation and loneliness. The average net promoter score (NPS) was 8.67 out of 10. Qualitative feedback was provided by 766 (10.79%) participants which amounted to 898 total responses. Most themes were rated as positive (517), followed by neutral (311) and a minor portion as negative (70). The following five themes were found across the qualitative responses: positive outcome (277, 30.8%), user did not address question (262, 29.2%), bonding with the chatbot (240, 26.7%), negative technical aspects (70, 7.8%), and ambiguous outcome (49, 5.5%). Themes with a positive valence were found to be associated with a higher NPS. The word “help” and it's variations were found to be the most frequently used words, which is consistent with the thematic analysis. These results show that a chatbot for social isolation and loneliness was perceived positively by most participants. More specifically, users were likely to personify the chatbot (e.g., “Cause I feel like I have a new friend!”) and perceive positive personality features such as being non-judgmental, caring, and open to listen. A minor portion of the users reported dissatisfaction with chatting with a machine. Implications will be discussed.

## Introduction

Social isolation has affected people globally during the COVID-19 pandemic and this can have a major impact on people's well-being (1). Prior to the COVID-19 pandemic, loneliness was already named an epidemic for older adults in the United States in 2017 by the US Surgeon General (2). Social isolation and loneliness can be hard to define as they are based on a subjective perception of relationships (2). Since the presentation of loneliness differs from person to person, there is a need to develop customizable interventions for specific groups based on needs and the degree of loneliness a person identifies (3). One systematic review on social isolation in older adults found that digital interventions were most efficacious in reducing loneliness and social isolation (4). A systematic review on interventions for reducing social isolation among people with mental health problems found that, overall, there is not strong evidence for interventions, however, there is promising evidence for interventions that include cognitive modification and those that support socialization (5). Only one study was found on a chatbot intervention for social isolation (6), however, the purpose of this study was not to assess if users had a sense of companionship with the chatbot and did not intend to measure the reduction of loneliness. Chatbot interventions may be a way to provide individualized, asynchronous support to address people's need to have someone to talk to.

There are several mental health and well-being chatbots that have been developed and are used commercially including Kokobot (7), Replika (6), Shim (8), Tess (9, 10), Woebot (11), and Wysa (12). These chatbots have been shown to lead to improvements in mood and symptoms of mental disorders. Few robust studies include analysis on what users think of the experience of using a chatbot, but there are no reported studies on chatbot interventions for social isolation.

### Perception of Mental Health Chatbots by Users

Chatbots have been perceived as humans and having human-like personalities since the first chatbot, Eliza. This is a Rogerian style chatbot designed to mirror the users who interacted with it (13, 14). There have been few studies that focused on understanding user's perceptions of chatbots. One scoping review identified ten themes across 37 unique chatbot studies (15). The themes were usefulness, ease of use, responsiveness, understandability, acceptability, attractiveness, trustworthiness, enjoyability, content, and comparisons.

Personality and identity attributes assigned to chatbots by the users' perceptions, or the developers has an impact on how people interact with them. Borau and colleagues (14) found that female chatbots are perceived as more human than male chatbots. They found that female chatbots are perceived to be human and more likely to respond to the needs of users. This should be considered in the development of future chatbots and the benefits and detriments of gendering them (16).

In a study conducted by Shumanov and Johnson (15) it was found that chatbots can be created in a way to express desirable personality traits. They concluded that users have higher engagement with chatbots that have similar personalities to the user. In this study, chatbot personality was defined dichotomously as extraverted and introverted. They developed two versions of the chatbot such that the extroverted chatbot's responses were assertive and commanding while the introverted chatbot used language that prioritized being efficient and goal oriented. It was found that matching extroverted individuals with extroverted chatbots or introverted individuals with introverted chatbots yielded a higher engagement compared to non-matched styles. Additionally, overall, introverted individuals engaged with the chatbot more than extroverts, which could indicate that introverts may be more accepting of machine interactions than extroverts.

A study on the chatbot Replika showed that users build relationships with the chatbot (17). It was found that users identified the chatbot as non-judgmental and were willing to disclose personal information to it and did not see any identified social risks. They also found that, overall, the relationship between the user and the chatbot was found to be rewarding and that they characterized the chatbot as accepting, understanding, and non-judgmental. A limitation of this study is that it included only 18 participants.

A study by Ta and colleagues (6) included a thematic analysis of user reviews of the Replika app and detailed open-ended responses to a self-reported survey on the chatbot. They found that four main themes emerged related to different types of support users perceived: informational support, emotional support, companionship support, and appraisal support. Additionally, two negative themes were observed; “uncanny valley” (users stated that the AI was “weird” or “creepy”) and out-of-place messages. Finally, there was one theme indicating no impact or users being unsure of the impact of the chatbot. Due to the fact that users identified the chatbot as a source of support, Ta and colleagues (6) state that chatbots may be a way to reduce loneliness in users through companionship. One limitation of this study is that demographic information is not known for users who provided the public reviews and the survey respondents included only 66 participants.

Prakash and Das (18) also conducted a study using publicly available user reviews on the chatbots Wysa and Woebot. They found four primary themes emerged from the reviews: perceived risks, perceived benefits, trust, and perceived anthropomorphism. Within the theme of perceived anthropomorphism, they identified several subthemes related to qualities the chatbot had including empathy, intelligence, and personality. Prakash and Das (18) identified that this anthropomorphism has positive and negative implications based on how human the users perceived the chatbot to be. They found that some users felt the chatbot was too human which led to feeling judged and increased anxiety (18).

Fitzpatrick and colleagues (11) conducted a study on the chatbot Woebot for young adults. This study included qualitative analysis of user's responses to questions on what users thought was best and worst about their experiences with the chatbot. Thematic analysis revealed two themes for the best parts of the chatbot: process and content. The process theme included comments on the chatbot's accountability, empathy, other “personality” features, the chatbot's facilitation of learning, and the conversation. Negative themes identified included issues with process violations, technical problems, and dissatisfaction with the content. It is noteworthy that many users gendered the chatbot and called it a “friend” despite the purposefully robotic name assigned to the Woebot to highlight that it was non-human.

However, several studies reported concerns about negative aspects of the chatbots including the chatbot misunderstanding users which led to frustration when irrelevant comments are provided (19). Additionally, most chatbot studies present several limitations. For example, they did not report on older adults. Terp and colleagues (20) found that when older adults perceived that the digital intervention was not designed for them or was not appropriate, they were less likely to engage with it and perceive benefits from it (20). Previous chatbot studies have measured user experience in a number of ways including asking multiple scaling questions related to how helpful the chatbot was, how much the users enjoyed their experience, and how satisfied the users are with the chatbot (10–12, 21). The types of questions used in past studies include heterogeneous quantitative measures, so it is hard to compare the results. Reichheld (22) proposed that developers use the net promoter question to assess the overall impression about a product. The net promoter question asks: “How likely is it that you would recommend our company to a friend or colleague.” It was found that this question is impactful because it identified how many users are willing to put their reputation behind a product.

Overall, specific impressions of a chatbot for adults and older adults, designed for social isolation and using the net promoter score (NPS) has not been reported.

### Current Study

It is important to consider that not all chatbots are equal in their development and past qualitative studies have posed a variety of questions to users in multiple different methods which may be a cause for heterogeneity in the findings. Net promoter questions are considered reliable measures of user satisfaction in multiple industries including customer service, education, and entertainment among others (23). The current study seeks to understand user experiences through the use of a net promoter question directed at current users of the chatbot and by requesting qualitative elaborations on why users provided a certain score.

The aims of the current study are to (1) understand the distribution of net promoter scores, (2) conduct a thematic analysis on qualitative elaborations to the net promoter scores, (3) understand the distribution of net promoter scores per theme, and (4) conduct a single word analysis to understand the frequency of words present in the qualitative feedback. It is hypothesized that high net promoter scores will be found, and the topics of the thematic analysis would be around usefulness, technical problems, and anthropomorphizing the chatbot.

## Method

### Participants

This was an open study and there were no inclusion and exclusion criteria beyond being an adult who lives in the US and Canada. Participants were recruited through Facebook advertisements that included content regarding social isolation and loneliness. There were 7,099 participants who consented to be part of the chatbot portion of this study. Participants engaged in a chatbot intervention from August 2019 to February 2020, which included assessments gathered at pre- and posttest, asynchronous engagement with the chatbot, and the opportunity to provide qualitative feedback. The results of the assessments are reported elsewhere (24) and the results of the qualitative feedback will be presented here.

### Materials

The materials for this study include a version of the chatbot (Tess) designed for adults and older adults. Tess is a mental health chatbot that uses an AI-based computer program to engage with users to teach coping skills and provide support. Users can engage with Tess interventions through text message conversations or Facebook Messenger. Other studies provide more information on how Tess works (9). Recruitment advertisements were delivered via Facebook stating that the chatbot intended to reduce social isolation, loneliness, and depression. The advertisement included the text, “are you feeling lonely? Coach [Tess] can help you.”

### Measures

The measures in this study are two questions posed by the chatbot. The first is the NPS question: “Ok, using a 0–10 scale: How likely is it that you would recommend me to a friend or colleague?” Participants were then asked to type a numerical response. The second question was posed after the user responded to the NPS question which will be called the qualitative question: “If you don't mind me asking, what was the primary reason for your score?”

### Procedures

Users expressed their interests by initiating the conversation with the chatbot via Facebook Messenger. They were then sent an introductory message which explained what a chatbot is and included a link to the chatbot's privacy policy and a consent form. To access these forms, users needed to click on the links which directed users out of Facebook messenger. If participants agreed, they were directed back to the Facebook Messenger conversation to begin interacting with the chatbot.

Feedback questions were embedded within the chat for those participants who interacted with the chatbot consistently. After the chatbot's algorithm determined that the users engaged sufficiently they were sent the NPS question (see Measures section). The user's response to this question was coded as their net promoter score. Next, the chatbot asked the participant to elaborate and asked the qualitative question (see Measures section). The user's response to this question was considered their qualitative response. Participants who engaged with the chatbot throughout the data collection period were offered the net promoter question multiple times thus could have multiple net promoter scores and qualitative responses. The data for this study was collected by the chatbot development company and de-identified prior to being shared with the research team. The study was deemed as non-human subjects research by the Institutional Review Board at Palo Alto University (Assurance Number: FWA00010885).

### Coding Strategy

An iterative thematic analysis was used to identify themes in user's responses to the elaboration on the net promoter question using Braun and Clarke's (25) method for thematic analysis in psychology. First, two authors (GD and EB) reviewed all the responses and identified the themes that were present in each. This open coding resulted in 25 themes, which were then discussed and merged. At this stage, comments were organized into categories which represented more than one theme and by valence (positive, negative, or neutral). The preliminary codes were reduced to 4 broad categories with 10 themes across the categories. It was determined that comments could be coded in more than one category unless they were assigned the theme “Hard to code.” A code book was developed with definitions created by the authors. Examples of the five themes were selected after the definitions were established.

Next, a graduate student was provided training on the codes and definitions. They were blinded to the code assignment initially given and were asked to code all user comments based on the code book. Since the interrater reliability was low, the authors reviewed the codes identified by the graduate student and reviewed all items for which there was not a match. After considering the items for which consensus was not reached, certain themes were merged, and others were divided. The authors also assigned new names to the themes at this stage and eliminated the categories. At this point, there were five themes that were assigned a positive, negative, or neutral valence. The revised code book with the themes, definitions, and examples was then given to another blind coder. An acceptable interrater reliability was achieved between the second blind coder and the authors.

### Analysis Plan

The distribution of NPSs was calculated by determining the frequency of each potential score (0–10) based on the score each user provided the first time they were offered the net promoter question.

Inter-rater reliability between the coders was assessed using Cohen's (26) kappa, which is frequently used to assess agreement between coders on categorical variables. Kappa statistics between 0.00 and 0.20 indicated slight agreement, 0.21 to 40 indicated fair agreement, 0.41 to 0.60 indicated moderate agreement, 0.61 to 0.80 indicated substantial agreement, and 0.81 to 1.00 indicated almost perfect agreement (26).

The qualitative response data was cleaned to make all letters lowercase, remove punctuation (quotation marks, commas, periods), and remove extra spaces between words. A data frame was created presenting a list of each word used in the qualitative responses across participants with the number of times it appeared. A list of stopwords created by Fox (27) was used to remove words that make poor indexing terms.

## Results

Of the 7,099 users who consented to be part of the chatbot portion of the study, 777 (10.95%) unique users responded to the NPS question and 766 (10.79%) of these users elaborated with a qualitative explanation of this score. Demographic characteristics of the 777 participants that answered the NPS question are presented in Table 1. Due to a large portion of users not providing demographic information, two percentages are presented: percentage of overall sample and percentage of those who provided demographic responses. Based on the participants that provided demographic information, the majority identified as female (*N* = 481, 88.26%). The largest age group was those ages 55–60 (*N* = 230, 42.28%). Most people identified as White (*N* = 323, 65.92%) and reported not living alone (*N* = 328, 63.08%). The most participants reported having a high school diploma or equivalent as the highest level of education (*N* = 219, 42.86%), being unable to work (*N* = 214, 29.63%), divorced (*N* = 168, 30.88%), and having an annual income of $0-$14,999 (*N* = 230, 51.92%).

**Table 1:** Demographic characteristics.

	***N***	**% of overall sample**
**Gender**		
Female	**481**	**61.90**
Male	64	8.24
Other	0	0
Decline to State	232	29.86
**Age**		
Under 55	135	17.37
55–60	230	29.60
61–65	95	12.23
66–70	43	5.53
71–75	27	3.47
76–80	8	1.03
80 and older	6	0.77
Decline to state	**233**	**29.99**
**Ethnicity**		
Asian	15	1.93
Black	51	6.56
White	**323**	**41.57**
Latin American	14	1.80
Indigenous	21	2.70
Mixed	24	3.09
Other	23	2.96
Do not know	19	2.45
Decline to State	287	36.94
**Living Status**		
Living Alone	192	24.71
Not Living Alone	**328**	**42.21**
Decline to State	257	33.08
**Education**		
Schooling but not a HS diploma	117	15.06
HS diploma/equivalent	**219**	**28.19**
College certificate or diploma, trade, vocational or technical school	126	16.22
University	23	2.96
PhD or equivalent	1	0.13
Other	21	2.70
Do not know	4	0.51
Decline to state	266	34.23
**Employment status**		
Employed, full time	57	7.34
Employed, part time	41	5.28
Unemployed and currently looking for work	51	6.56
Unemployed and not currently looking for work	23	2.96
Student	3	0.39
Retired	104	13.38
Volunteer	3	0.39
Homemaker	27	3.47
Self-employed	17	2.19
Unable to work	214	27.54
Decline to state	**237**	**30.50**
**Marital status**		
Never married	89	11.45
Married	117	15.06
Separated	62	7.98
Divorced	168	21.62
Widowed	108	13.90
Decline to state	**233**	**29.99**
**Income**		
0–14,999	230	29.60
$15,000–$19,999	61	7.85
$20,000–$24,999	36	4.63
$25,000–$29,999	24	3.09
$30,000–$34,999	9	1.16
$35,000–$39,999	6	0.77
$40,000–$59,000	18	2.32
Over $60,000	13	1.67
Do not know	46	5.92
Decline to state	**334**	**42.99**

*Bolded means in the table represent the largest subgroups*.

### Net Promoter Score Distribution

**Figure 1:**
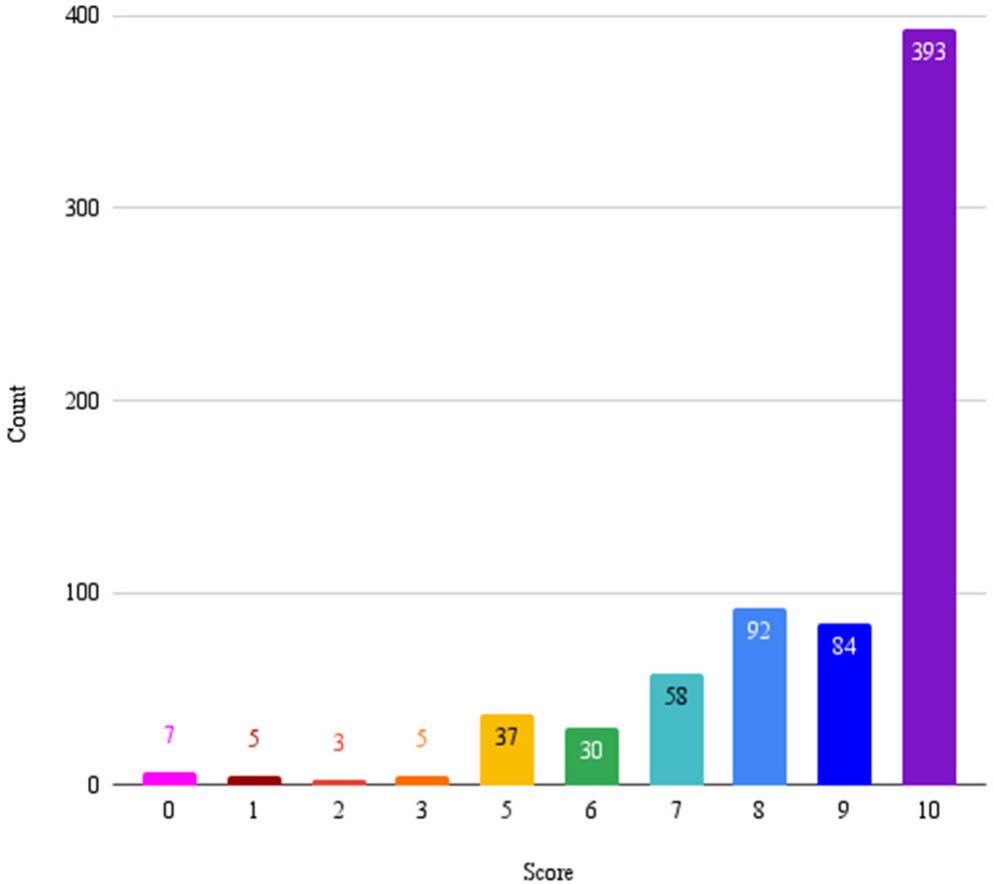
Each bar represents the number of times each possible NPS score was given by users.

The NPS question is set to be delivered 4 days after the first conversation and then a second NPS inquiry is sent 47 days after that. There were a total of 1,050 net promoter scores given by the 777 unique users. After removing duplicates (multiple responses from a single participant) and non-numerical responses, a total of 720 users responded to the NPS question with a score between 0 and 10. The distribution of NPS scores is presented in Figure 1. The mean NPS was 8.67 (*SD* = 2.05), fifty-seven people gave scores that were non-numerical responses. Most users gave an NPS of 10 (*N* = 393, 50.58%) followed by 8 (*N* = 92, 11.84%), and 9 (*N* = 84, 10.81%). The least common net promoter score was 2 (*N* = 3, 0.39%).

### Thematic Analysis

**Figure 2:**
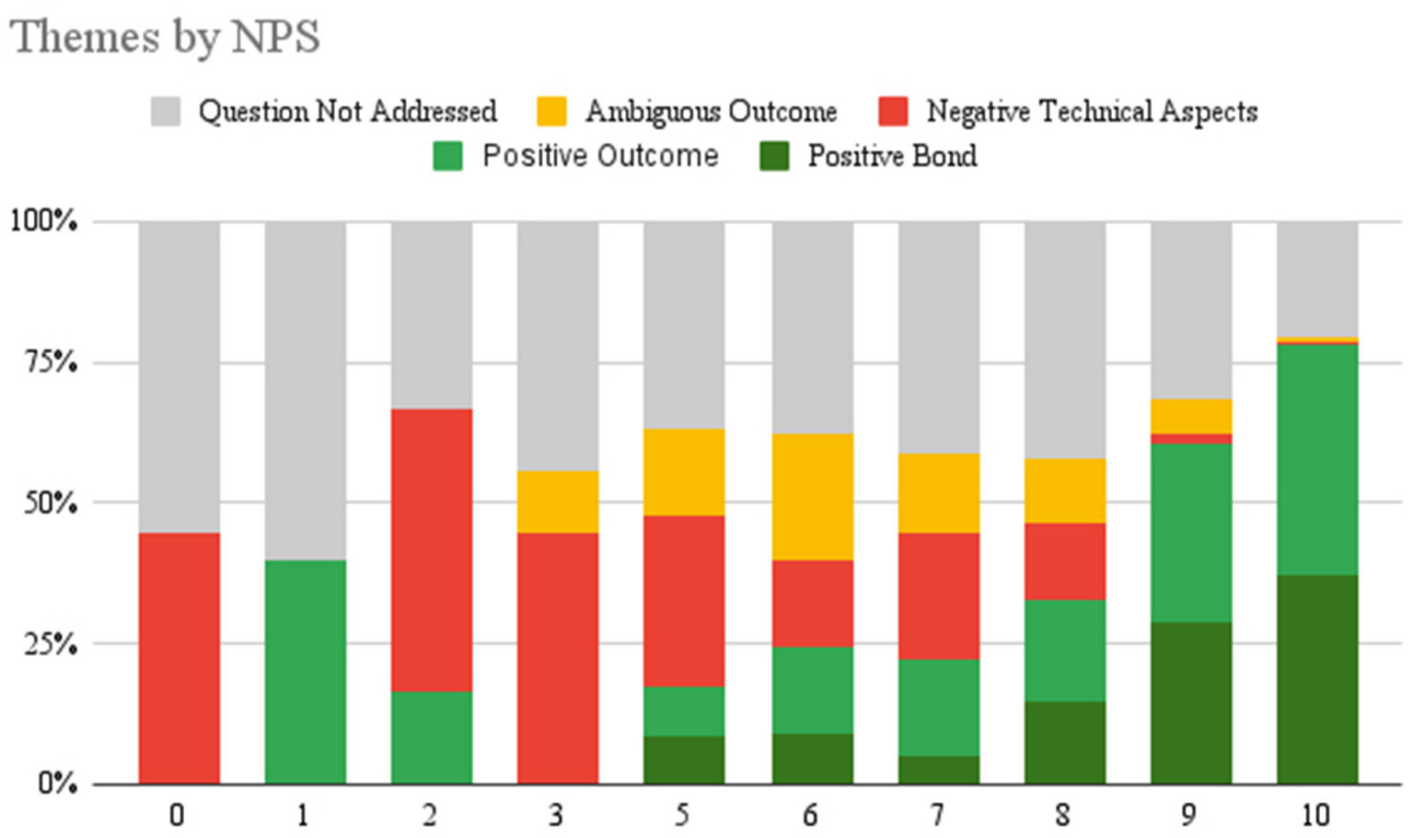
Each NPS score provided which was associated with a qualitative elaboration is presented here to show the distribution of themes by each NPS score.

**Figure 3:**
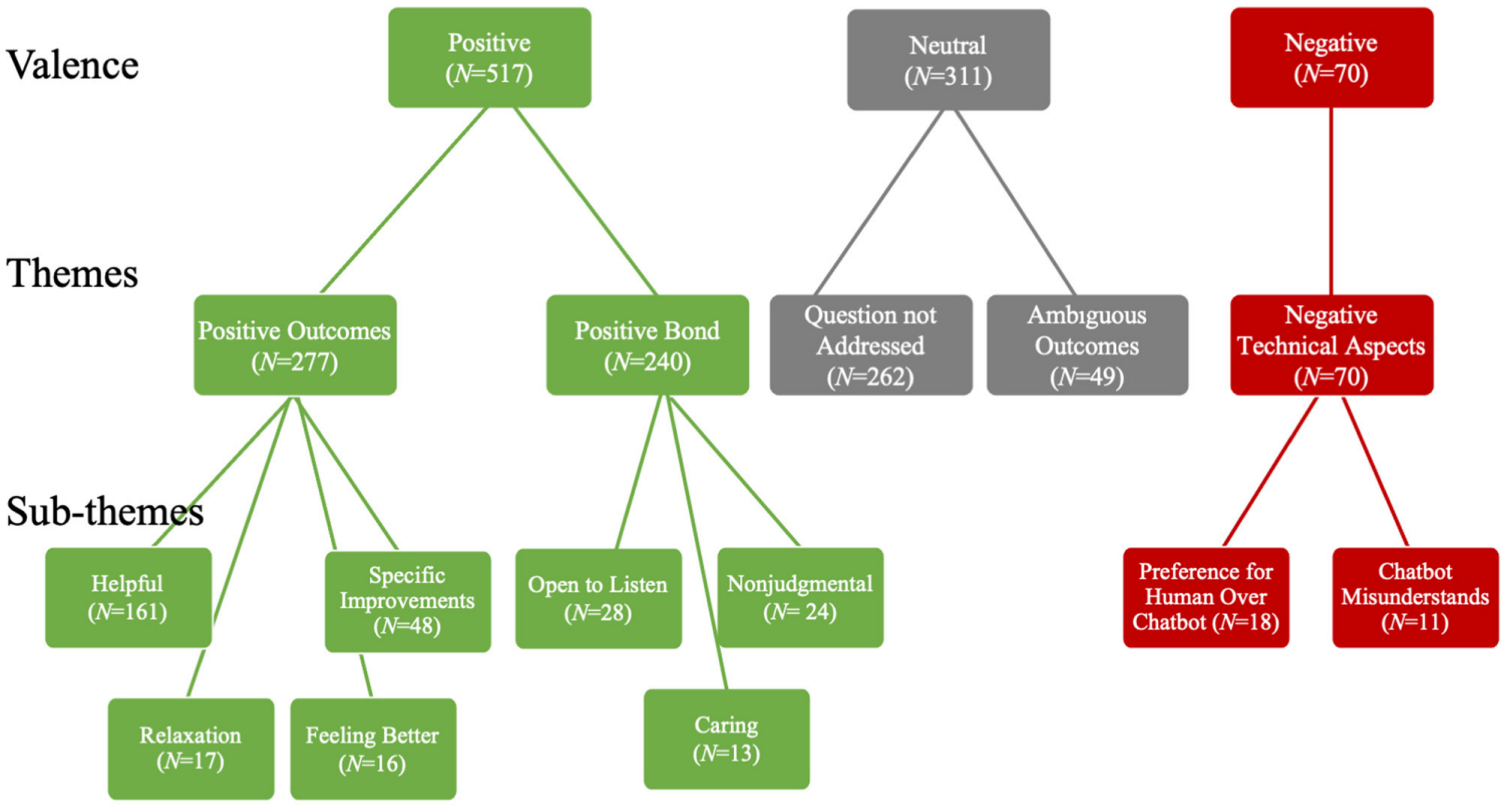
Organization and number of user comments for each valence, theme, and sub-theme.

Qualitative feedback was provided by 766 (10.79%) participants which amounted to 898 total responses. There were five themes identified. The most frequent theme was Positive Outcome, followed by Question Not Addressed, Positive Bond, Negative Technical Aspects, and Ambiguous Outcomes. See Table 2 for definitions, examples, frequencies, and interrater reliabilities of each theme. Additionally, these themes were grouped by valence: positive, neutral, negative; and broken down into sub-themes. For a visual representation of the thematic analysis see Figure 2. Examples of each sub-theme are presented below. Figure 3 presents the number of user responses for each valence, theme, and sub-theme.

**Table 2:** Themes, definitions, examples, frequencies, and interrater reliability.

**Themes**	**Definitions**	**Examples**	**Frequency (%)**	**Kappa**
Positive Outcome	The user stated that talking with the chatbot was helpful. The user stated that their symptoms improved, their presenting concern was addressed, talking to the chatbot helped their mood improve or to relax. The user said that they feel better. The user stated that they learned something from the chatbot including new coping skills or other tips.	“Because every time we have a conversation I feel better & more relaxed afterwards.” “Because this is going to potentially save my life and the lives of many others.” “Because you have skills to help people to stay calm.”	277 (30.8)	0.78
Question Not Addressed	The user's response does not seem to relate to the NPS question, or the answer is not clear.	“I've got to go do some thing, I'll talk with you later.” “No real reason.” “I don't usually recommend things to people.”	262 (29.2)	0.82
Positive Bond	The user reported a positive rapport (harmonious relationship with the chatbot and felt understood) or that the chatbot had a positive personality (e.g., non-judgmental, open to listen, or caring).	“You deserve it you good kind person we need more like you.” “This is great therapy for me. I can express myself without judgment, without stereotyping me or my situation U have not once put me down or pointed a finger in any negative way, so I thank u for all of help.” “Cause I feel like I have a new friend!”	240 (26.7)	0.86
Negative Technical Aspects	The user expressed that there are miscommunications or that the chatbot responds in a vague way that does not always relate to the issue the user brought up. The user stated that it does not feel like talking to a real person or that it is not equivalent to face-to-face services. The user stated they feel uncomfortable talking to a chatbot or machine.	“First, you should remember me when I log in a[n]d second, you should remember our previous conversations and be more interactive with me.”“The conversation feels clinical at times and you know its not a human responding.”“I don't always feel like you understand the point I'm trying to express.”	70 (7.8)	0.86
Ambiguous Outcome	The user expressed that they did not know how they felt about the chatbot. Some stated that the intervention has been too short, or they need more time or information to determine if it will be helpful.	“I am ambivalent about our interactions.”“I think the score will go up as we go along and you learn and evolve. I do like your style of “speaking”… warm, but not overly familiar or fake.”	49 (5.5)	0.57
Overall				0.81

#### Positive Outcomes

Of the 277 comments in the Positive Outcome theme, 161 qualitative responses were on the chatbot's helpfulness. Some users commented that the chatbot was helpful (e.g., ““You helped me to write down concerns and know maybe somebody will help and listen to me”). There were 48 comments indicating a specific improvement (e.g., “These conversations are teaching me to think about my feelings or thoughts to take action to improve myself”). Some users commented that the chatbot helped them gain coping skills in general (e.g., “So far you have some great ideas for me to try something new to try to cope with what I'm going through,” other participants mentioned that the chatbot helped them learn specific coping skills such as relaxation strategies (*N* = 17; e.g., “You have helped me to learn how to breathe right to relax through the stress and it really helps”). Finally, 16 comments included reports of feeling better since interacting with the chatbot: (e.g., “Because every time we have a conversation I feel better & more relaxed afterwards”).

#### Positive Bond

The positive bond theme was broken down into three sub-themes: open to listen, non-judgmental, and caring. Twenty-eight comments mentioned that the chatbot was open to listening or was a good listener (e.g., “You truly listened to me and didn't just say get over it”; “I enjoy you reaching out to me knowing someone is always there to listen or help”; “You listened to me when nobody else would”). There were 24 comments on the chatbot being perceived as non-judgmental (e.g., “I feel that you understand without judging”). Thirteen comments were on the chatbot being perceived as caring (e.g., “You seem to genuinely care” and “Because you seemed really concerned and caring”).

#### Negative Technical Aspects

Two sub themes were identified for the Negative Technical Aspects theme including a preference for human over chatbot interactions and the chatbot misunderstanding the user. Eighteen comments described a preference for talking or chatting with humans over chatbots [e.g., “A human looks at the person and can tell by body language and facial expressions about what is going on. Typing on a keyboard doesn't…”; “It's not like writing with a human”; “Because although I think (you are) helpful I wouldn't say (you are) a total 10 because (you are) not a real person”]. There were 11 comments regarding the chatbot misunderstanding or providing vague responses [e.g., “Sometimes you don't (sic) comprehend what I am saying and I get irritated”; “You need to pay attention to what the other person says”; “Your responses are very general - not specific to my particular situation”].

### Themes by NPS

For each NPS the themes associated with the elaborations were analyzed. A total of 482 people provided an NPS score of 10 and elaborated on their response, the most common themes were positive outcome (*N* = 197, 40.9%), positive bond (*N* = 179, 37.1%), and question not addressed (*N* = 99, 20.5%). Theme distributions for other scores are presented in Figure 2 below.

### Single Word Analysis

To better understand the frequency of specific words in the qualitative responses, a word analysis was conducted. A total of 1,239 unique words were found based on the 766 qualitative responses of varying lengths. After removing the stopwords from Fox's (27) stopword list, 957 words remained. When the stopwords were excluded, a total of 2,618 words were included in the analysis.

The most common word to appear was “help,” which was stated 94 times. Variations on the word help such as “helpful,” “helped,” “helps,” “helping,” appeared 55, 43, 13, and 11 times, respectively. The word help and variations of this word appeared a total of 203 times representing 7.75% of the total words used. A list of all words and frequencies is presented in Appendix A.

Though “you” is one of the stopwords included in Fox's list (27), it is noteworthy that this word appeared in the qualitative responses 384 times and was the second most common word following “I” and “to.” It was used in such phrases as “you are non-judgmental,” “because I feel you truly understand me,” and “I can count on you in the future.” The construction of these comments show that users are responding to the chatbot as if it were a person rather than a machine. This personification is significant as it shows that this chatbot may have passed the Turing test, though users had been made aware that they were interacting with a machine.

## Discussion

Social isolation has affected people globally during the COVID-19 pandemic and had a major impact on an individual's well-being (28). Chatbot interventions may be a way to provide support to address loneliness and social isolation. Chatbots have been shown to lead to improvements in mood and symptoms of mental disorders (6–8, 10–12). However, research on chatbots is in the early stages, there are no studies on chatbots for social isolation, and understanding how individuals perceive the chatbot can inform future developments.

The current study analyzed the responses of a sample of mostly white females with about a third of the participants identifying as a racial or ethnic minority. The sample was recruited through advertisements targeting individuals feeling lonely and a considerable portion of the participants reported living alone (24.7%) and only 15% were married, so these results are representative of a specific set of the population and the results may not be generalizable to people who do not feel lonely.

### NPS

Regarding the NPS a total of 720 users responded to the NPS question, the mean NPS was 8.67 and a considerable high proportion of users gave an NPS of 10 (*N* = 393, 50.58%), 9 (*N* = 84, 10.81%), and 8 (*N* = 92, 11.84%). One previous study on a chatbot for problematic gambling reported a NPS of−33 which was determined by subtracting the number of detractors from the number of supporters (29). This study did not report the raw scores of participants thus it is not possible to compare it to the scores obtained in the present study. Other chatbot studies did not utilize NPS, however they did assess user satisfaction through other means. Fitzpatrick and colleagues found that on average users reported high levels of satisfaction (4.3/5) and high self-reported levels of increased emotional awareness after using the chatbot (3.3/5) (11). In another study, a thematic analysis indicated that 67.7% of responses found that interacting with the chatbot was favorable (12). Fulmer and colleagues (10) found that 86% of their sample reported being overall satisfied with their experience with the chatbot. Vaidyam and colleagues (21) found that overall, users were satisfied with interacting with a chatbot and scored it above 4.2 out of 5 on a number of parameters.

These results indicate that satisfaction with the chatbot in this study may be comparable or higher than past studies on chatbots for adults. However, it is not known how much users engaged with the chatbot prior to providing feedback. Studies on user engagement and feedback report differences in findings based on whether users are asked the NPS question soon or later in their interactions. When the NPS or satisfaction question is asked sooner, it is associated with lower scores (12, 20). In this study how much the users engaged with chatbot prior to receiving this NPS question was not considered.

To note, the NPS question was offered only to participants who reached the final section of specific conversations, and only 11% of the sample provided the NPS thus it is unclear how the remaining 89% of the sample perceived the chatbot. It is possible that some engaged positively with the chatbot but not enough to reach the NPS questions or that they have been dissatisfied with the chatbot and stopped utilizing it. Thus, a major portion of user experiences remain unknown.

### Thematic Analysis

Congruent with other chatbot studies, a thematic analysis was used to further understand user experiences (10–12). To further the understanding of how users perceived the chatbot in this study, the explanations to the NPS were analyzed with a thematic analysis. Qualitative feedback was provided by 766 (10.79%) participants which amounted to 898 total responses. The most frequent theme was Positive Outcome, followed by Question Not Addressed, Positive Bond, Negative Technical Aspects, and Ambiguous Outcomes.

#### Positive Outcomes

Regarding Positive Outcomes, there were over 160 comments on the chatbot being helpful in some way to users. Some users commented that the chatbot was helpful, associated with specific improvements, helped them gain general or specific coping skills, and several users reported feeling better since interacting with the chatbot. This indicates that chatbots can provide some benefit to users who are open to using them and may be an effective way to provide basic support to users including teaching relaxation and coping skills. These specific improvements were referenced by several users as being effectively taught by the chatbot. Some of these themes were also observed in a study conducted by Fitzpatrick and colleagues (11) (i.e., checking in/accountability, empathy/personality, and learning). Fulmer and colleagues (10) also found accessibility, empathy, and learning as elements of the chatbot that users enjoyed most, which is similar to the findings of the current study.

#### Question Not Addressed

The second most common theme was Question Not Addressed. Since the chatbot was addressing individuals experiencing loneliness and the NPS question asks about how likely users are to recommend the chatbot to a friend, some responded to this question by stating they do not have friends. For those who do have friends, they may be concerned about how other people would perceive their user-chatbot relationship. Skjuve and colleagues (17) found that most chatbot users were hesitant about sharing their relationship with a chatbot with other people due to social stigma. One user in the present study reported that they are “not sure anyone else would want to talk to a non-person about their feelings.” Another user reported “being nervous about friend or colleague thinking I'm crazy for talking to a machine.”

#### Positive Bond

The third most common theme was the relationship users established with the chatbot. This theme reflects how individuals can relate to an AI chatbot and assign personality traits to it such as being caring, open to listen, and non-judgmental. This is consistent with past studies that found that users identified a mental health chatbot as non-judgmental and were willing to disclose personal information to it and did not see any identified social risks of doing so (17).

Within the Positive Bond theme, users talked about how the chatbot is open to listen and is available. This highlights that chatbots are asynchronous and may be available to users when traditional psychotherapy is not, such as over the weekend or at night, which indicates that chatbots are an accessible mental health resource. Future studies could aim to understand when users engage with the chatbot to assess if they are being used when traditional psychotherapy is unavailable. Interestingly, several comments referenced the COVID-19 pandemic. Data collection for this study ended in February 2020 and since some participants were older adults, they may have already been experiencing self-isolation. One user reported “Because through this stay at home order it is easy to get worked over simple things and you have helped me get through a lot of things.” This shows that chatbots may be a source of support when face-to-face interventions are not possible.

Many users referred to the chatbot as “you” rather than “it” or “the chatbot” which indicates that they are personifying it. Several individuals used gendered pronouns to describe the chatbot including one who identified the chatbot's sex as a benefit: “You truly made me feel so much better & that your [sic] a female!” This raises ethical dilemmas about the potential representation of a chatbot as a person. Though chatbot developers often take care to highlight that chatbots are non-human, sometimes users still perceive them as human as was also found in other studies on human perception of chatbots (11).

#### Negative Technical Aspects

While the Negative Technical Aspects theme was found in <10% of responses, it still shows that chatbots have limitations. Some users commented on chatbot misunderstandings which is consistent with past studies that have found that users expressed frustration when chatbots misunderstand questions and give irrelevant responses (19). This highlights the need to improve chatbot development including training of AI algorithms.

Other comments indicated a preference for human over chatbot interactions. This shows that users who are not interested in a digital intervention may not be the target population for chatbot interventions. This indicates that not everyone is well-suited for chatbots, assessing affinity toward chatbots interventions in the early stages may help identify those who could benefit from other referral options. In previous research it has been found that some users have a difficult time connecting with a chatbot because they perceive it as too human so this may be an additional explanation for resistance to using a chatbot (18). Fulmer and colleagues (10) found that users also reported that the chatbot was impersonal, misunderstood the user, and was too general. This highlights the need for ongoing improvements in the technical aspects of chatbots.

### Theme by NPS and Single Word Analysis

Since the themes were created manually and include human bias two additional analyses were conducted: a distribution analysis of themes by NPS and a single word analysis.

There was a consistency between scores given and themes reported in qualitative responses. Lower NPS were associated with more negative themes and users not addressing the question and higher scores were associated with positive valence themes. This provides further support for the themes created and their associated valence.

The single word analysis showed that the word “help” and its variations were the most common words to appear in the qualitative responses. This is consistent with the most commonly found theme, Positive Outcome, which included a helpfulness sub-theme. Overall, the combination of both a thematic analysis and single word analysis provides an additional layer of support for the themes discussed. From all the studies on chatbots reviewed (10–12, 17), none have used a single word analysis. This is the first chatbot study that conducted a more objective analysis after the thematic analysis to capture the most relevant themes as reported by the users.

### Limitations

There were several limitations in the current study. First, demographic information is missing for about 30% of the sample. Participants were asked to provide demographic information, but were not required to, so many participants gave qualitative feedback without providing some demographic information. This reduces the generalizability of the overall findings. Second, there was limited diversity in the sample so future studies should aim to recruit a more diverse sample as far as gender identity, age, and ethnic background.

Third, asking individuals that identified as being lonely weather they would recommend this to a friend (i.e., NPS question) may not be the most appropriate question to assess their satisfaction with the chatbot. Since users may not have enough friends or feel embarrassed about sharing with others that they are chatting with a chatbot, finding alternative ways of assessing the bot are needed. Ryu and colleagues (30) stated that it is essential to the development of a mental health care chatbot that users are involved in the design and development process. Involving users in adapting the chatbot intervention could provide feedback on how to phrase the net promoter question to minimize user discomfort.

### Future Directions

There is heterogeneity in the type of questions and measures used across chatbot studies to assess satisfaction and helpfulness. This makes it difficult to compare chatbots and establish meaningful benchmarks of efficacy. Future studies should aim to use a standardized measure, such as the NPS, which may make it easier to compare chatbots. With this in consideration, researchers should be considerate of when the wording of the NPS question should be amended or adapted to best suit the sample of the study.

One limitation in the current study is that there are many comments that could not be interpreted due to typos or non-sensical writing. Since the current sample included older adults, one possible explanation for the large amount of non-sensical writing could be related to Ryu and colleagues (30) finding that for older adults it can be difficult to type on a keyboard due to issues with vision or dexterity. One potential solution, proposed by Ryu and colleagues (30), for this when designing chatbots for older adults is to use buttons which can often be integrated into chatbots delivered in instant messaging services. Including buttons may be supported for chatbots for adults of all ages. Inkster and colleagues found that most users preferred to respond by clicking preformatted options (12). Utilizing buttons or giving sentence starters may be a way to increase feedback and reduce the likelihood of the feedback question being misunderstood.

Future studies aiming to understand large qualitative datasets should aim to use more advanced natural language processing techniques. Though the word “help” and its variations appeared a large number of times in the current data set, from a single word analysis it is not possible to tell when these words were used in a negative context such as saying “not helpful.”

In previous research it has been found that some users have a difficult time connecting with a chatbot because they perceive it as too human so this may be an additional explanation for resistance to using a chatbot (18). Developers should consider this and strive to balance anthropomorphizing the chatbot and highlighting that it is not human so that users do not feel judged.

## Conclusion

Social isolation and other mood problems affected people globally and have been intensified by the COVID-19 pandemic (2). There is a need to develop interventions to address these issues and chatbots may be a way to provide individualized and asynchronous support to people who are open to it. The results of this study largely support previous findings that show that most chatbot users tend to be satisfied and would recommend it to a friend or colleague. The results indicate two themes of dissatisfaction with the chatbot emerged. The first one indicates there is still a need to improve the development of mental health chatbots to reduce misunderstandings. The second one shows there is a portion of users that do not feel comfortable talking to a chatbot. Most interestingly, results show a pattern of personifying the chatbot and assigning human traits to it such as being helpful, caring, open to listen, and non-judgmental. Additionally, it was found that from asynchronous and exclusively text-based conversations users were able to build a bond with a chatbot.

## Data Availability Statement

The raw data supporting the conclusions of this article will be made available by the authors, without undue reservation.

## Author Contributions

All authors listed have made a substantial, direct and intellectual contribution to the work, and approved it for publication.

## Conflict of Interest

The authors declare that the research was conducted in the absence of any commercial or financial relationships that could be construed as a potential conflict of interest.

## Publisher's Note

All claims expressed in this article are solely those of the authors and do not necessarily represent those of their affiliated organizations, or those of the publisher, the editors and the reviewers. Any product that may be evaluated in this article, or claim that may be made by its manufacturer, is not guaranteed or endorsed by the publisher.
