# Supplementary material for: Bonding With Bot: User Feedback on a Chatbot for Social Isolation
Source: Front Digit Health. 2021 Oct 6;3:735053. doi: 10.3389/fdgth.2021.735053 (PMC8526729; doi:10.3389/fdgth.2021.735053)
Supplement: Supplementary file 1 [file Table_1.pdf]

Appendix A
Results of Single Word Analysis

Word	Frequency
Help	94
Feel	87
Don’t	79
Talk	72
Helpful	55
Helped	43
I’m	43
People	40
It’s	31
Time	29
10	25
Talking	25
Understand	25
Am	21
Listen	21
Person	21
Ok	20
Cause	19
Lot	19
You’re	17
Feeling	16
Friends	16
Advice	14
Easy	14
Nice	14
Score	14
That’s	14
Helps	13
Anxiety	12
&	11
Didn’t	11
Enjoy	11
Helping	11
Little	11
Tell	11
Yes	11
A lot	10
Else	10
Sometimes	10

Note. Other words with frequencies lower than 10 included care, love, real, depressed, listening, positive, relax, and understanding.
